# TECHNOLOGY-DRIVEN INTERGENERATIONAL PROGRAM IN THE PANDEMIC

**DOI:** 10.1093/geroni/igac059.3065

**Published:** 2022-12-20

**Authors:** Sheryl Groden, Matthew Carpenter, Jennifer Blackwood, Allon Goldberg

**Affiliations:** University of Michigan-Flint, Flint, Michigan, United States; University of Michigan-Flint, Flint, Michigan, United States; University of Michigan-Flint, Flint, Michigan, United States; University of Michigan-Flint, Flint, Michigan, United States

## Abstract

Intergenerational programs increase interaction among groups allowing both generations to share their beliefs, talents, knowledge, and wisdom. The Senior Programming Intergenerational College Experience (SPICE) Project was designed to deliver an 11-week long intergenerational program with college-aged and older adult participants and was provided virtually during the pandemic. The purpose of this study was to describe the participation characteristics and attitudes towards aging and technology of older adults who completed the SPICE program. Older adult recruitment occurred through paper fliers posted at senior centers/housing, libraries, faith-based organizations in Genesee County, Michigan and through a large state-wide electronic study recruitment portal. Older adult participants were English speaking; aged 55+ years; able to participate in live Zoom sessions once/week; and were committed to complete >75% of the SPICE program. Online surveys were completed before/after SPICE to evaluate attitudes towards aging and technophilia. Results from 15 older adults who completed >75% of SPICE sessions indicated that they were able to cope with life as they got older (60%), viewed aging as a positive experience (85.7%), were not afraid to use new technology (68.6%) and believed technology was useful/fun (88.6%). Older adults were divided on willingness to try new technology and their ability to keep up with technological progress. These data indicate that for higher education institutions focused on designing age-inclusive online opportunities, older adults who possess a positive attitude toward aging and technology may be more likely to complete intergenerational programs.

